# Polycyclic Aromatic Hydrocarbons (PAHs) and Metals in Diverse Biochar Products: Effect of Feedstock Type and Pyrolysis Temperature

**DOI:** 10.3390/toxics11020096

**Published:** 2023-01-19

**Authors:** Hattan A. Alharbi, Khaled D. Alotaibi, Mohamed H. EL-Saeid, John P. Giesy

**Affiliations:** 1 Department of Plant Protection, College of Food and Agriculture Sciences, King Saud University, P.O. Box 2460, Riyadh 11451, Saudi Arabia; 2 Department of Soil Science, College of Food and Agriculture Sciences, King Saud University, P.O. Box 2460, Riyadh 11451, Saudi Arabia; 3 Toxicology Centre, University of Saskatchewan, Saskatoon, SK S7N 5B3, Canada; 4 Department of Integrative Biology, Michigan State University, East Lansing, MI 48824, USA; 5 Department of Environmental Sciences, Baylor University, Waco, TX 76798, USA

**Keywords:** biochar, chicken manure, sewage sludge, olive pomace, feather meal, soft meal, date palm residues

## Abstract

Biochar’s agricultural and environmental benefits have been widely demonstrated; however, it may cause environmental contamination if it contains large amounts of pollutants such as polycyclic aromatic hydrocarbons (PAHs) and heavy metals (HMs). Therefore, this study aimed to assess the contents of PAHs and HM in a range of biochars generated from different sources and pyrolysis temperatures. A range of feedstock was converted to biochar, including sewage sludge (SS), olive mill pomace (OP), feather meal (FM), soft offal meal (CSM), chicken manure (CM), and date palm residues (DPR). Each feedstock was then pyrolyzed at three temperatures of 300, 500, or 700 °C, thereby producing a total of 18 types of biochar. These biochar products were analyzed for 16 PAHs and eight metals (Cr, Mn, Fe, Ni, Cu, Zn, Cd, and Pb). Benzo[b]fluoranthene, benzo[k]fluoranthene, and benzo(a)pyrene were significantly greater in the biochar produced at 700 °C than in that produced at 300 °C, especially for CM. The concentrations of dibenz(a,h)anthracene were significantly lower at 700 °C but greater at 500 °C and 300 °C in DPR. Increasing the pyrolysis temperature from 300 to 700 °C significantly increased the concentrations of metals, including Cr in SS and OP; Mn in CM; and Fe, Ni, Cu, and Zn in SS. However, the concentration of Cd was significantly lower in the SS when biochar was produced at 700 °C than at 500 or 300 °C. The type of feedstock used and the pyrolysis temperature are key factors influencing the contents of PAHs and HMs in biochar, both of which need to be considered during the production and use of biochar. Further investigations are recommended to establish the relationships between pyrolysis temperature and types of feedstock and the formation of PAH or the concentrations of metals. Monitoring the concentrations of PAHs and HMs before applying biochar to soil is also recommended.

## 1. Introduction

Biochar is considered an effective soil amendment with the potential to improve many physical, chemical, and biological attributes of soils [1,2], which can increase the crop yields by providing better availability of nutrients and a greater potential for soils to retain water [3,4,5]. Biochar also plays a major role in mitigating abiotic stresses in plants, for example, the treatments of plants with biochar might increase plants resistant to heat-induced stress [6]. It may therefore be used as a tool in environmental management [7]. However, regardless of the potential benefits, biochar also contains variable concentrations of toxic elements, such as heavy metals (HM), volatile organic compounds (VOCs), and polycyclic aromatic hydrocarbons (PAHs), in addition to dioxins [8]. The concentrations of these chemical components, however, largely vary, depending upon the feedstock type and pyrolysis conditions (such as residence time, temperature, and heating rate) [9,10]

The initial composition of the feedstock substantially influences the content of metals and other toxic components in the resulting biochar. For instance, sewage sludge has a relatively high concentration of metals [9,11,12], and during pyrolysis, metals can undergo volatilization, enrichment, and speciation in the residues [13]. These metals become a part of the soil solution and exchange sites when biochar is applied as an amendment to the soil [8]. According to the International Biochar Initiative (IBI), thresholds of metals in biochar include Pb (Min = 121; Max = 300), Cd (Min = 1.4; Max = 39), Cu (Min = 143; Max = 6000), Ni (Min = 47; Max = 420), Hg (Min = 416; Max = 7400), Cr (Min = 93; Max = 1200), Co (Min = 34; Max = 100), Mo (Min = 5; Max = 75), and Se (Min = 2; Max = 200) at mg/kg dry weight [14]. However, the ecological risk of metals in biochar could be decreased if feedstock such as sewage sludge is pyrolyzed at an appropriate temperature [15]. The ecotoxicity of metals largely depends on the leachability and mobility of metals from biochar [16,17]

The PAHs are persistent organic contaminants that have two or more aromatic rings, the exposure to which induces potential risks and hazards to the environment [18]. Furthermore, PAHs can also adversely affect animals and humans, considering they are mutagenic, carcinogenic, and teratogenic [19,20]. The application of biochar with large concentrations of PAHs to soil additionally increases the potential for releasing these organic contaminants into the environment, particularly resulting in soil and plant contamination, thereby increasing the risk to public health [21,22].

The production of PAHs is mostly associated with the incomplete combustion of fossil fuels [19]; however, the partial aerobic combustion of raw biomass for biochar production under high temperatures (pyrolysis) can also result in the production of PAHs [10]. It has been hypothesized that recondensation during pyrolysis produces several (semi-) volatile organic compounds [23,24,25]. In addition, fluctuations in temperature, the composition of raw feedstock, and other analytical conditions, such as pressure, determine the type and concentration of PAHs [26]. The concentrations of PAHs in biochar, which can be coproduced during pyrolysis, may pose a potential risk to the environment [27,28,29,30].

It has also been observed that higher temperatures (1000 °C) and anaerobic conditions produce a small amount of syngas (~20%) and a large quantity of bio-oil (~60% of mass) and PAHs [23,24]. However, under low-temperature pyrolysis, relatively greater amounts of biochar (ca. 35–50%) and fewer PAHs are produced [31]. It has been shown that the type and chemical composition of feedstock can affect the amounts and types of PAHs in the produced biochar [29,32,33]. The amounts of lignin, C, H, N, and O elemental contents and cellulose and hemicellulose determine the quality and quantity of PAHs [31,34]. Concentrations of PAHs in biochar range from as low as 100 µg kg^–1^ to 100 mg kg^–1^ and can be in the thousands of mg kg^–1^ for the sum (Σ) of the 16 US EPA PAHs [31,35].

Considering the increasing use of biochar in agricultural and environmental applications, it is important to evaluate the number of contaminants in biochar to ensure its quality and avoid negative effects on soils. Research on the quality of biochar is essential, especially when considering the wide range of organic materials (feedstock types) used for its production and the pyrolysis temperature, all of which are critical factors that influence the chemical composition of biochar. Further studies to investigate a wide range of biochars derived from various feedstocks at different temperatures for their content of contaminants are essential to determine the magnitude of variability among biochars according to their production-related factors. This study, therefore, aimed to assess the biochar content of PAHS and metals as a function of the feedstock type and pyrolysis temperature. It was hypothesized that the pyrolysis temperature and feedstock type would result in variations in the concentrations of PAHs and metals in biochar.

## 2. Material and Methods

### 2.1. Feedstock Collection and Biochar Production

Six organic materials were used as feedstocks for the production of biochar. These include (1) sewage sludge (SS), (2) olive mill pomace (OP), (3) feather meal (FM), (4) soft offal meal (CSM), (5) chicken manure (CM), and (6) date palm residues (DR). These materials were selected based on their local abundance and chemical composition. All six organic materials were converted into biochar at three pyrolysis temperatures—300, 500, and 700 °C. A sample of each feedstock was first homogenized and then placed in a tightly sealed stainless chamber to ensure oxygen-limited conditions. The feedstock was pyrolyzed in a muffle furnace at the desired temperature with a retention time of 3 h. The biochar produced was allowed to cool to room temperature, thoroughly mixed, ground manually to pass through a 1.0 mm sieve, and stored in glass jars in the lab until analysis. A total of 18 biochar products were obtained and evaluated in this study.

### 2.2. Extraction and Quantification of PAHs

Concentrations of 16 PAHs were determined in biochar, including 2-ring [naphthalene-(Nap)], 3-ring [acenaphthene (Ace)], acenaphthylene (Acy), fluorene (Flu), phenanthrene (Phe), anthracene (Ant)], 4-ring [fluoranthene (Fla)], pyrene (Pyr), chrysene (Chr), benzo[a]anthracene (BaA), 5-ring [benzo[b]fluoranthene (BbF)], benzo[k]fluoranthene (BkF), benzo[a]pyrene (BaP), dibenz[a,h]anthracene (DahA)], 6-ring [indeno-1,2,3-cd-pyrene (IcdP)], and benzo[ghi]perylene (BghiP)]. Extraction and quantification of PAHs were conducted following the procedure described by Hilber et al. 2012. Briefly, the PAHs were extracted using the Soxhlet extraction method with 100% toluene for 36 h [36]; after which, 1 g of dried and homogenized biochar was briefly added to the sleeves of the Soxhlet extractor for 36 h with toluene. After extraction, the volume of the solvent was reduced to 10 mL using a rotary vacuum evaporator and then to approximately 1 mL using nitrogen. The extract was then spiked with 15 μL of the recovery standard (250 ng acenaphthene-d10 in toluene) and concentrated to 1 mL using GC-MS. Targeted PAHs were identified and quantified using a Thermo Scientific™ TSQ 8000™ triple–quadrupole GC-MS/MS system, equipped with a Thermo Scientific™ TRACE™ 1310 GC with an SSL Instant Connect™ SSL module and Thermo Scientific™ TriPlus™ RSH autosampler. The injection mode was splitless, with a splitless injection volume of 1 μL and a time of 1.0 min. A DB-5MS GC column (30 m × 0.25 mm × 0.25 μm) was used. The carrier gas used was 99.99% pure He at a flow rate of 1.2 mL/min. The temperature program was set at 100 °C, 1 min; 10 °C/min to 160 °C, 4 min; and 10 °C/min to 250 °C, 2 min. The ionization mode used was EI with 70 eV, the ion source temperature was 250 °C, and single reaction monitoring was used with a transition setup automatically built up by Auto SRM software.

### 2.3. Quantification of Metals

The quantification of the total metal (HMs) content involved digesting 0.2 g of biochar with a mixture of HNO_3_, HCl, and H_2_O_2_ in a microwave digestion system (Jin et al. 2016 [9]; Udayanga et al. 2018 [13]). The digested samples were diluted with deionized water to 50 mL; after which, the concentrations of Cr, Fe, Ni, Cu, Zn, Cd, and Pb were determined using an ICP-MS instrument (Thermo Fisher Scientific GmbH, Bremen, Germany). An analysis was conducted on three replicates of each biochar type.

### 2.4. Statistical Analyses

Two-way ANOVA was used to examine the effects of feedstock type and pyrolysis temperature on the concentrations of PAHs and HMs, and the treatment means were compared using Fisher’s LSD. To understand the similarity and dissimilarity among PAH and HM concentrations along different feedstocks and pyrolysis temperatures, the datasets were statistically analyzed using a cluster analysis and principal component analysis (PCA) with SPSS (IBM-Statistical Package for the Social Sciences, version 22) software. The cluster analysis grouped the datasets into similar categories and demonstrated the homogeneity of the various PAHs and HMs among the different feedstocks.

Statistical significance was determined at a 95% confidence interval with a significance level of 0.05. Statistical analyses were performed using Origin 2021 Pro OriginLab Corporation (Northampton, MA, USA).

## 3. Results

### 3.1. Metals Concentrations

**Figure 1:**
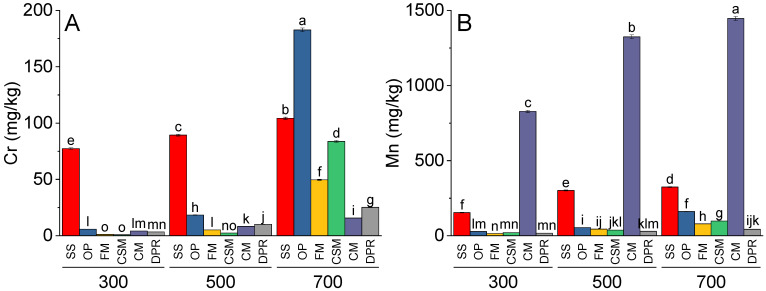

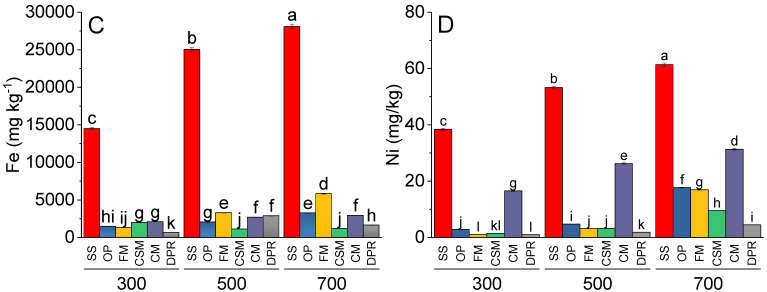
Effect of type of feedstock and pyrolysis temperature (300, 500, or 700 °C) on the concentrations of Cr (**A**), Mn (**B**), Fe (**C**), and Ni (**D**) in the biochars. Different color bars indicate the means of three replicates. Error bars represent ± standard error. Letters on bars represent a significant difference at *p* ≤ 0.05. SS = sewage sludge; OP = olive mill pomace; FM = feather meal; CSM = soft offal Meal; CM = chicken manure; DPR = date palm residues.

The effects of feedstock and pyrolysis temperature were significant for the concentrations of chromium (Cr), manganese (Mn), iron (Fe), and nickel (Ni) in biochar. At 300 °C and 500 °C, SS demonstrated significantly greater concentrations of Cr than OP, FM, CSM, CM, and DPR. Cr was also significantly greater in OP than in FM, CSM, CM, and DPR at 300 and 500 °C. At 700 °C, OP contained higher concentrations of Cr than SS, FM, CSM, CM, or DPR (Figure 1A). Compared to 300 and 500 °C, Cr was significantly greater at 700 °C in the SS (0.35 and 0.17-fold), OP (30.61 and 8.93-fold), FM (42.39 and 8.63-fold), CSM (90.89- and 34.25-fold), CM (2.74- and 0.90-fold), and DPR (6.49- and 1.49-fold). Concentrations of Mn were significantly greater in CM than in SS, OP, FM, CSM, and DPR at 300 °C, 500 °C, and 700 °C., and increasing the temperature from 300 °C to 700 °C resulted in higher concentrations of Mn in SS, OP, FM, CSM, and CM (Figure 1B). At 300 °C, 500 °C, and 700 °C, SS contained significantly higher concentrations of Fe than OP, FM, CSM, CM, or DPR. In OP and FM, the Fe concentration increased marginally at higher pyrolysis temperatures—that is, 300–500 and 700 °C; however, Fe was lower in CSM but greater in CM and DPR at 500 and 700 °C than at 300 °C (Figure 1C). The concentrations of Ni in SS and CM were significantly greater than those in OP, FM, CSM, and DPR at 300, 500, and 700 °C. A significant enhancement in Ni was also observed in OP, FM, CSM, and DPR when the pyrolysis temperature was increased from 300 to 500 and 700 °C (Figure 1D).

**Figure 2:**
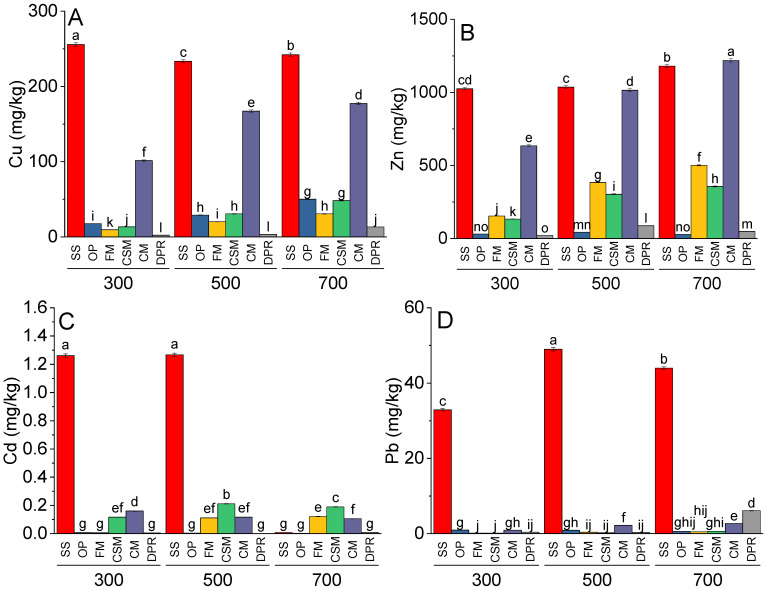
Effects of feedstock and pyrolysis temperature (300, 500, or 700 °C) on Cu (**A**), Zn (**B**), Cd (**C**), and Pb (**D**) in diverse biochars. Different bars indicate the means of three replicates. Error bars represent ± standard error. Letters on bars represent a significant difference at *p* ≤ 0.05. SS = sewage sludge; OP = olive mill pomace; FM = feather meal; CSM = soft offal Meal; CM = chicken manure; DPR = date palm residues.

The concentrations of Cu were significantly greater at 500 °C and 700 °C in the SS than at 300 °C. In OP, FM, CSM, and CM, higher production temperatures (500 and 700 °C) significantly enhanced Cu content compared to that at 300 °C (Figure 2A). No significant change in Cu was observed in the DPR pyrolyzed at 300 or 500 °C; however, the concentration of Cu was significantly greater at 700 °C than at 300 and 500 °C in the DPR. On average, the greatest amount of Cu was observed in the SS, followed by the CM. In FM, CSM, and CM, Zn was significantly greater at 500 °C and 700 °C compared to 300 °C. In DPR, concentrations of Zn were significantly greater at 500 °C, followed by 700 °C compared at 300 °C. In SS, Zn did not differ significantly at 300 and 500 °C but remained significantly greater at 700 °C (Figure 2B). The concentrations of Cd remained significantly greater in SS at 300 and 500 °C than 700 °C. For FM and CSM, concentrations of Cd were significantly greater at 500 and 700 °C compared to 300 °C. However, the concentrations of Cd in CM were significantly less at 500 and 700 °C compared to 300 °C (Figure 2C). Significantly more Pb was observed in the SS at 500 °C than at 300 °C; however, in SS, the concentration of Pb was significantly greater at 500 °C than at 700 °C. In the CM, the concentration of Pb was significantly greater at 500 and 700 °C than at 300 °C. In DPR, no significant difference was observed between the concentrations of Pb at 300 and 500 °C, but a significant enhancement was observed at 700 °C (Figure 2D).

### 3.2. Concentrations of PAHs in Biochar

**Figure 3:**
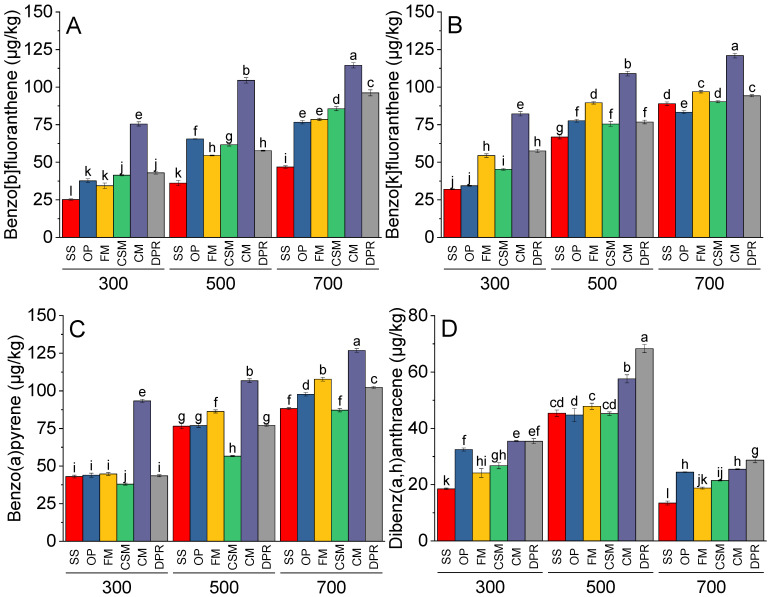
Effects of feedstock and pyrolysis temperature (300, 500 and 700 °C) on great molecular PAHs i.e., benzo[b]fluoranthene (**A**), benzo[k]fluoranthene (**B**), benzo(a)pyrene (**C**), and dibenz(a,h)anthracene (**D**) in the biochar samples. Different bars indicate the means of three replicates. Error bars represent ± SE. Letters on bars are representing a significant difference at *p* ≤ 0.05. SS = sewage sludge; OP = olive mill pomace; FM = feather meal; CSM = soft offal Meal; CM = chicken manure; DPR = date palm residues.

The concentrations of benzo[b]fluoranthene, benzo[k]fluoranthene, and benzo(a)pyrene increased in SS, OP, FM, CSM, CM, and DPR at 500 and 700 °C than at 300 °C. Compared to SS, OP, FM, CSM, and DPR, benzo[b]fluoranthene (Figure 3A), benzo[k]fluoranthene (Figure 3B), and benzo(a)pyrene (Figure 3C) were significantly greater in CM. Significantly more dibenz(a,h)anthracene was observed at 500 °C than at 300 °C.; however, at a pyrolysis temperature of 700 °C there was significantly less dibenz(a,h)anthracene formed than at 300 °C (Figure 3D). On average, CM and DPR exhibited the highest concentrations of dibenz(a,h)anthracene at 300 °C. The highest concentration of dibenz(a,h)anthracene was observed for the DPR pyrolyzed at 500 °C.

**Figure 4:**
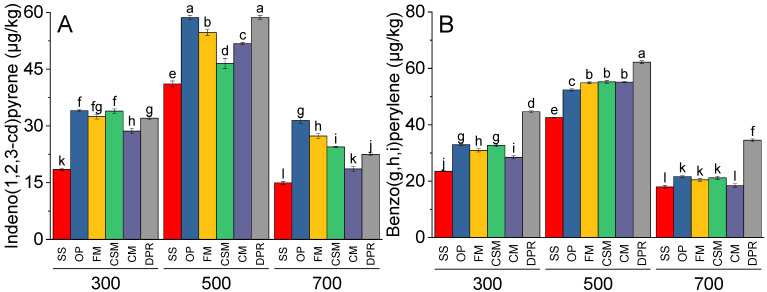
Effects of feedstock and pyrolysis temperature (300, 500 and 700 °C) on greater molecular mass PAHs i.e., indeno(1,2,3-cd)pyrene (**A**) and benzo(g,h,i)perylene) (**B**) in the biochar samples. Different bars indicate the means of three replicates. Error bars represent ± SE. Letters on bars are representing a significant difference at *p* ≤ 0.05. SS = sewage sludge; OP = olive mill pomace; FM = feather meal; CSM = soft offal Meal; CM = chicken manure; DPR = date palm residues.

Indeno(1,2,3-cd)pyrene (Figure 4A) and benzo(g,h,i)perylene (Figure 4B) differed significantly at 500 °C compared to 300 °C. Significantly less indeno(1,2,3-cd)pyrene and benzo(g,h,i)perylene) were observed at 700 °C than at 300 °C. At 300 °C, significantly more indeno(1,2,3-cd)pyrene was observed in the OP, FM, and CSM than in SS. However, at 500 °C, significantly more indeno(1,2,3-cd)pyrene was formed from CPR and OP than from SS. At 700 °C, the amount of indeno(1,2,3-cd)pyrene was significantly greater in OP than in SS. Benzo(g,h,i)perylene was significantly greater in DPR than in SS, OP, FM, CSM, and CM at 300 °C, 500 °C, and 700 °C.

**Figure 5:**
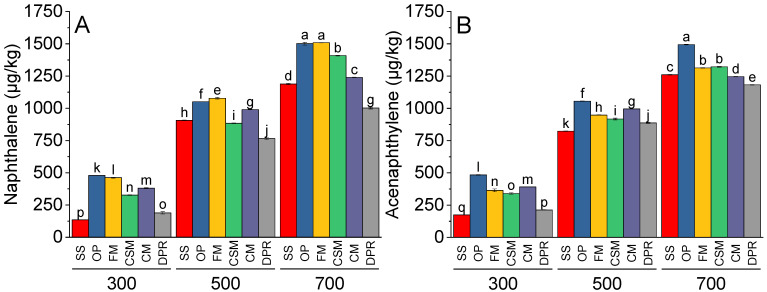

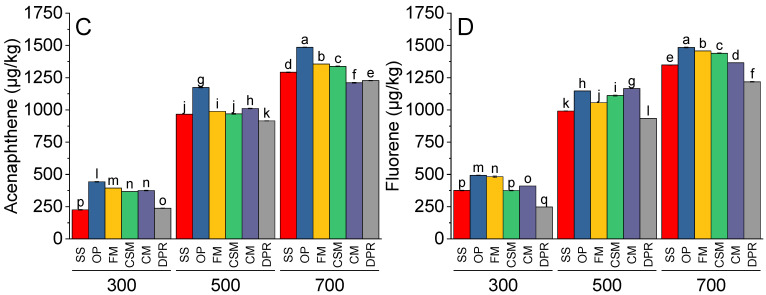
Effects of feedstock and pyrolysis temperature (300, 500, and 700 °C) on low molecular PAHs, i.e., naphthalene (**A**), acenaphthylene (**B**), acenaphthene (**C**), and fluorene (**D**) in the biochar samples. Different bars indicate the mean of three replicates. Error bars represent ± standard error. Letters on bars represent a significant difference at *p* ≤ 0.05. SS = sewage sludge; OP = olive mill pomace; FM = feather meal; CSM = soft offal Meal; CM = chicken manure; DPR = date palm residues.

Concentrations of naphthalene (Figure 5A), acenaphthylene (Figure 5B), acenaphthene (Figure 5C), and fluorene (Figure 5D) were significantly greater at 500 and 700 °C than at 300 °C. The naphthalene concentration was greatest in the OP at 300 °C and FM at 500 °C. At 700 °C, the OP and FM contained similar concentrations of naphthalene, which were greater than those in SS, CSM, CM, and DPR. The concentrations of acenaphthylene, acenaphthene, and fluorene were higher in OP than in SS, FM CSM, CM, and DPR at 300, 500, or 700°.

**Figure 6:**
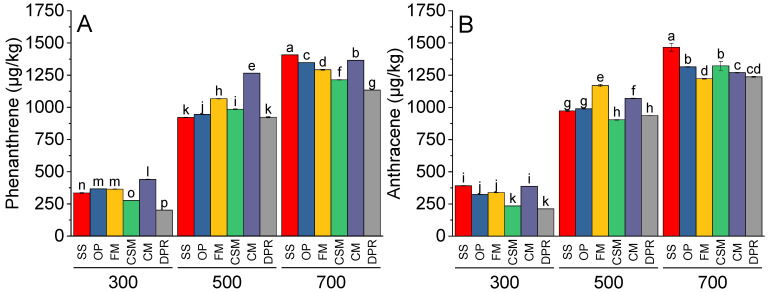

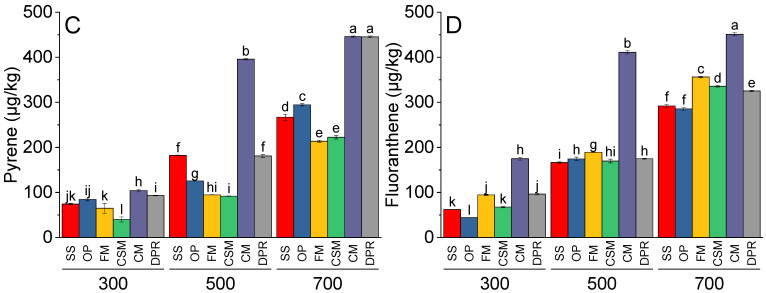
Effects of feedstock and pyrolysis temperature (300, 500, and 700 °C) on low molecular PAHs, i.e., phenanthrene (**A**), anthracene (**B**), pyrene (**C**), and fluoranthene (**D**) in the biochar samples. The different bars indicate the means of three replicates. Error bars represent ± standard error. Letters on bars represent a significant difference at *p* ≤ 0.05. SS = sewage sludge; OP = olive mill pomace; FM = feather meal; CSM = soft offal Meal; CM = chicken manure; DPR = date palm residues.

**Figure 7:**
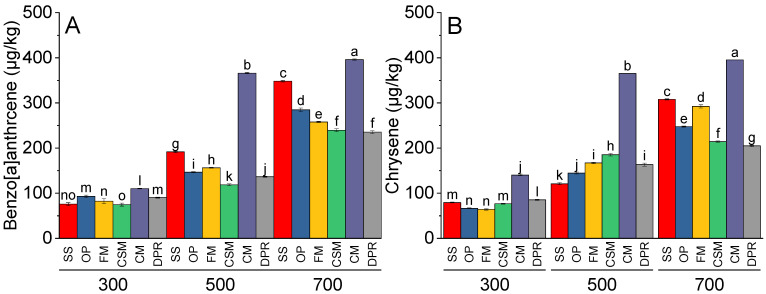
Effects of feedstock and pyrolysis temperature (300, 500, and 700 °C) on low molecular PAHs, i.e., benzo[a]anthrcene (**A**) and chrysene (**B**) in the biochar samples. The different bars indicate the means of three replicates. Error bars represent ± standard error. Letters on bars represent a significant difference at *p* ≤ 0.05. SS = sewage sludge; OP = olive mill pomace; FM = feather meal; CSM = soft offal Meal; CM = chicken manure; DPR = date palm residues.

The production of phenanthrene (Figure 6A), anthracene (Figure 6B), pyrene (Figure 6C), and fluoranthene (Figure 6D) was directly proportional to the pyrolysis temperature. The concentrations of phenanthrene were the highest in CM at 300 °C and 500 °C. At 700 °C, SS contained greater amounts of phenanthrene than did FM, CSM, CM, and DPR. The concentrations of anthracene were similar in the biochars prepared from SS and CM, with maximum production at 700 °C. At 500 °C, the anthracene concentrations in FM were greater than those in SS, CSM, CM, and DPR. However, the concentrations of anthracene were significantly greater in SS at 700 °C, while the concentrations of pyrene and fluoranthene were significantly greater in CM at 500 and 700 °C; however, at 700 °C, the concentrations of pyrene were similar in DPR and CM. Only CM demonstrated maximum concentrations of fluoranthene formed at 700 °C. At 500 and 700 °C, benzo[a]anthracene (Figure 7A) and chrysene (Figure 7B) were significantly greater than those produced at 300 °C. At 300, 500, and 700 °C, the concentrations of benzo[a]anthracene and chrysene were highest in CM compared to SS, CSM, CM, and DPR.

**Figure 8:**
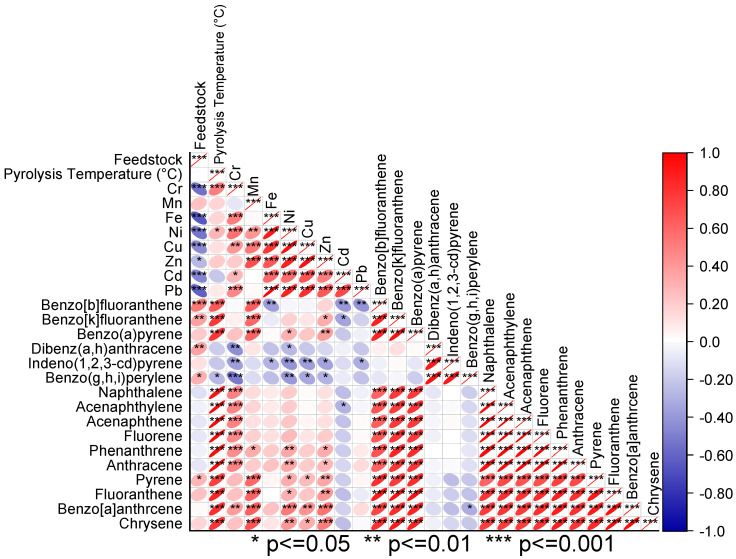
Pearson’s correlation for biochar feedstock, pyrolysis temperature, metals, and PAHs. Red represents positive correlations while blue represents negative correlations. The intensity of color demonstrates the significance of correlation.

**Figure 9:**
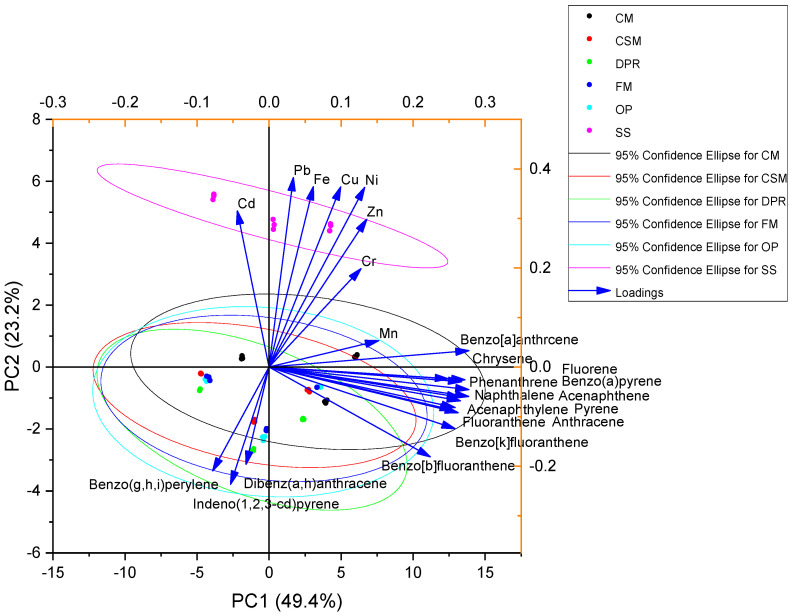
Principal component analysis of metals and PAHs using pyrolysis temperature as observation and feedstock as a group.

The analysis of Pearson’s correlation showed that feedstocks were significantly negatively correlated with the concentrations of Cr, Fe, Ni, Cu, Zn, Cd, and Pb, as indicated in Figure 8. However, a significant positive correlation was observed among the feedstocks for benzo[b]fluoranthene, benzo[k]fluoranthene, dibenz(a,h)anthracene, benzo(g,h,i)perylene, and pyrene. The concentrations of Cr, benzo[b]fluoranthene, benzo[k]fluoranthene, naphthalene, acenaphthylene, acenaphthene, fluorene, phenanthrene, anthracene, pyrene, and fluoranthene were significantly and positively correlated (Figure 8). Principal component analysis revealed that Cr, Fe, Ni, Cu, Zn, Cd, and Pb were associated with SS. However, the PAHs were not associated with the feedstocks (Figure 9).

## 4. Discussion

Variations were observed in the concentration of metals in the biochar depending on the feedstock type and pyrolysis temperature. These results indicated that metals might be present in the parent feedstock material from which biochar was produced, but it depends on feedstock type. The higher concentrations of heavy metals in biochar are due to the partial mineralization of organic substances during the pyrolysis process. For example, the concentration of heavy metals were higher than that in the feedstock sewage sludge [11]. The enrichment of heavy metals in biochar enhanced with the pyrolysis temperature, and the average metal concentration was found to be proportional to the pyrolysis temperature. Although the concentrations of heavy metals are higher in biochar than its feedstock, the toxicity and bioavailability of heavy metals associated with biochar is lower than its feedstock [11]. During pyrolysis, the disintegration of organic compounds in feedstock tends to increase the concentrations of metals that do not degrade [37,38] Furthermore, most feedstocks from which biochar is produced are enriched with hydroxides, oxides, and sulfides. The provision of thermal stability to hydroxides, oxides, sulfides, and their salts plays an important role in the significant increases in the concentrations of metals as a function of temperature during pyrolysis [13]. Higher temperatures during pyrolysis also enhanced the diffusion kinetics inside the ash and reactions with an ash matrix. Such diffusion-trapped metals with mineral ash enhance the retention of metals, thus increasing the content of metals in biochar [39,40,41]. Similar increases in the concentrations of metals were also observed in the current study when the pyrolysis temperature was increased from 300 to 700 °C.

The concentrations of PAHs were also directly proportional to the temperature during the pyrolysis of biochar produced from various feedstocks. Higher temperatures during pyrolysis resulted in a greater production of volatile gases. Greater re-condensation of volatile organic compounds on the surfaces of biochar results in greater concentrations of PAHs in biochar [25]. Based on this result, the most of biochar produced from different feedstock are not recommended to use as a soil amendment. The concentrations of high molecular weight PAHs are higher than the maximum permissible content of Σ16 PAHs (US EPA) in biochar which is in ranges from 6 to 300 mg/kg [14]. During pyrolysis, higher temperatures initiate carbonization and aromatization. Both processes facilitate PAH synthesis during biochar production [10,35]. However, it has been shown that lower concentrations of PAH are produced during slow pyrolysis rate than during fast pyrolysis [35]. Unimolecular cyclization, dealkylation, dehydrogenation, and aromatization of feedstock lignin and cellulose result in the emission of H_2_O, CO_2_, CH_4_, and H_2_S [42]. These native compounds leave behind aromatized structures, which become low-molecular-weight PAHs during pyrolysis [43] and are the key factors responsible for the synthesis of PAHs [10,32,33,44,45]. However, the relationship between PAH concentration and the conditions under which biochar is produced is not well understood [35]. A previous study demonstrated greater concentrations of PAHs when the pyrolysis temperature was increased from 300 to 600 °C [39]. PAHs with greater toxic potency, such as benzo[a]pyrene and benzo[a]anthracene, become enriched in biochar when the pyrolysis temperature is greater than 500 °C [28]. The results of the current study are also consistent with previous findings, where concentrations of benzo[a]pyrene were significantly greater when biochar was produced from various feedstocks at 500 or 700 °C than at 300 °C. Chemical feedstocks consist of cellulose, hemicellulose, and lignin, all of which can produce PAHs in biochar. A feedstock rich in cellulose and pectin produces significantly more PAHs than lignin-enriched feedstock [10,46]. Finally, it has been suggested that biochar with high PAHs concentrations might not pose significant ecotoxicological effects on soil dwelling organisms when biochar mixed with soil but not when biochar directly evaluate its toxicity (i.e., without soil) [47].

## 5. Conclusions

Both the feedstock and pyrolysis temperature affected the concentrations of PAHs and HMs in the biochar. Biochar produced from SS, OP, and CM demonstrated higher concentrations of HM. Similarly, biochars of SS and CM have a greater potential to produce PAHs than other feedstocks. Low-temperature pyrolysis produces fewer PAHs and HMs in biochar. Further investigations are recommended to better establish relationships between temperature and feedstock and PAH formation and HM contents. Monitoring these contaminants after the application of biochar to soil is additionally necessary to ensure environmental safety.

## Data Availability

The dataset generated in this study can be obtained from the corresponding author upon reasonable request.
